# Comprehensive Analysis Reveals the Molecular Features and Immune Infiltration of PANoptosis-Related Genes in Metabolic Dysfunction-Associated Steatotic Liver Disease

**DOI:** 10.3390/biology14050518

**Published:** 2025-05-08

**Authors:** Yan Huang, Jingyu Qian, Zhengyun Luan, Junling Han, Limin Tang

**Affiliations:** 1 Medical College, Yangzhou University, Yangzhou 225000, China; 2 Taizhou School of Clinical Medicine, The Affiliated Taizhou People’s Hospital of Nanjing Medical University, Taizhou 225300, China

**Keywords:** PANoptosis, Metabolic dysfunction-associated steatotic liver disease, immune infiltration, bioinformatics

## Abstract

Background: Metabolic dysfunction-associated steatotic liver disease (MASLD), a chronic inflammatory disorder characterized by alcohol-independent hepatic lipid accumulation, remains poorly understood in terms of PANoptosis involvement. Methods: We integrated high-throughput sequencing data with bioinformatics to profile differentially expressed genes (DEGs) and immune infiltration patterns in MASLD, identifying PANoptosis-associated DEGs (PANoDEGs). Machine learning algorithms prioritized key PANoDEGs, while ROC curves assessed their diagnostic efficacy. Cellular, animal, and clinical validations confirmed target expression. Results: Three PANoDEGs (SNHG16, Caspase-6, and Dynamin-1-like protein) exhibited strong MASLD associations and diagnostic significance. Immune profiling revealed elevated M1 macrophages, naïve B cells, and activated natural killer cells in MASLD tissues versus controls. Further experiments verified the expression of the key PANoDEGs. Conclusions: This study provides new insights for further studies on the pathogenesis and treatment strategies of PANoptosis in MASLD.

## Simple Summary

Metabolic dysfunction-associated steatotic liver disease (MASLD) is a common liver condition caused by excessive fat buildup, not linked to alcohol, which can lead to inflammation and long-term damage. While a specific type of cell death called PANoptosis is suspected to play a role in MASLD, exactly how this happens remains unclear. This study used advanced computer analysis and lab experiments to identify PANoptosis-related genes and their possible involvement in MASLD progression. Researchers analyzed genetic data from patient samples and found three critical genes—SNHG16, Caspase-6, and DNM1L—that are significantly tied to MASLD progression. These genes could help diagnose the disease early. This study also discovered that certain immune cells, like inflammatory macrophages, are more active in MASLD patients, worsening liver damage. Tests in cells, animals, and human samples confirmed these findings. By revealing how PANoptosis contributes to MASLD, this research offers new clues for developing treatments that target these genes or immune responses, potentially improving liver health for millions affected by this condition.

## 1. Introduction

Metabolic dysfunction-associated steatotic liver disease (MASLD), previously known as nonalcoholic fatty liver disease (NAFLD), is a clinico-histopathologic entity with histologic features that resemble alcohol-induced liver injury, but, by definition, it occurs in patients with little or no history of alcohol consumption. It encompasses a histologic spectrum that ranges from fat accumulation in hepatocytes without concomitant inflammation or fibrosis (simple hepatic steatosis) to hepatic steatosis with a necroinflammatory component (steatohepatitis) that may or may not have associated fibrosis. The latter condition, referred to as metabolic dysfunction-associated steatohepatitis (MASH), previously known as nonalcoholic steatohepatitis (NASH), may progress to cirrhosis in up to 20% of patients. MASH is recognized as a leading cause of cryptogenic cirrhosis [1,2].

The pathogenesis of MASLD has not been fully elucidated. Genetic factors, like PNPLA3 gene variants, increase susceptibility. The most widely supported theory implicates insulin resistance as the key mechanism leading to hepatic steatosis and perhaps also to steatohepatitis. Others have proposed that a “second hit”, or additional oxidative injury, is required to manifest the necroinflammatory component of steatohepatitis. Obesity, hepatic iron, gut hormones, antioxidant deficiencies, and intestinal bacteria have all been implicated in the pathogenesis of MASLD. Key points in the management of MASLD include weight loss through nutritional and lifestyle interventions, treatment of metabolic coexisting conditions, risk stratification for liver disease with the use of noninvasive tests, liver-directed pharmacotherapy, and management for advanced liver disease, with consideration of social and commercial health implications [3,4].

Increasing evidence suggests that programmed cell death (PCD), including pyroptosis, apoptosis, and necroptosis, is involved in MASLD [5,6,7,8]. PANoptosis is a form of PCD characterized by the integration of pyroptosis, apoptosis, and/or necroptosis [9,10]. However, PANoptosis is a distinct process that cannot be defined solely by any one of these modes of cell death. The process is mediated by specific triggers that activate the formation of the PANoptosome complex, leading to an inflammatory PCD pathway [11,12]. We compiled a table summarizing the characteristics of these PCD (Supplementary Table S1). Absent in melanoma 2 (AIM2), pyrin, and Z-nucleic-acid binding domain protein 1 (ZBP1) interact to assemble with an apoptosis-associated speck-like protein containing a C-terminal CARD (ASC), caspase-1, and receptor-interacting serine/threonine-protein kinase 3 (RIPK3) to form the PANoptosome, driving the process of PANoptosis [13]. Studies have indicated that AIM2 and activated caspase-1 protein levels are elevated in MASLD. Mitochondrial DNA (mtDNA) activates the AIM2 inflammasome in the liver of MASLD mouse models [14]. Notably, the NLR family pyrin domain containing 3 (NLRP3) inflammasome plays a significant role in the mechanisms underlying MASLD and liver fibrosis [15,16]. Moreover, studies have indicated that hepatic RIPK3 is closely associated with MASLD severity in both humans and mice and plays a critical role in managing liver metabolism, injury, inflammation, fibrosis, and carcinogenesis [17]. Thus, hypothesizing that MASLD is closely associated with PANoptosis is reasonable.

In recent years, advancements in high-throughput sequencing technologies and bioinformatics analyses have been increasingly utilized to explore potential molecular mechanisms and targeted therapies for various diseases, paving the way for future clinical research [18,19]. To the best of our knowledge, few studies have investigated the role of PANoptosis in the pathogenesis of MASLD, and no relevant genes associated with PANoptosis have been reported in bioinformatics research on MASLD.

**Figure 1:**
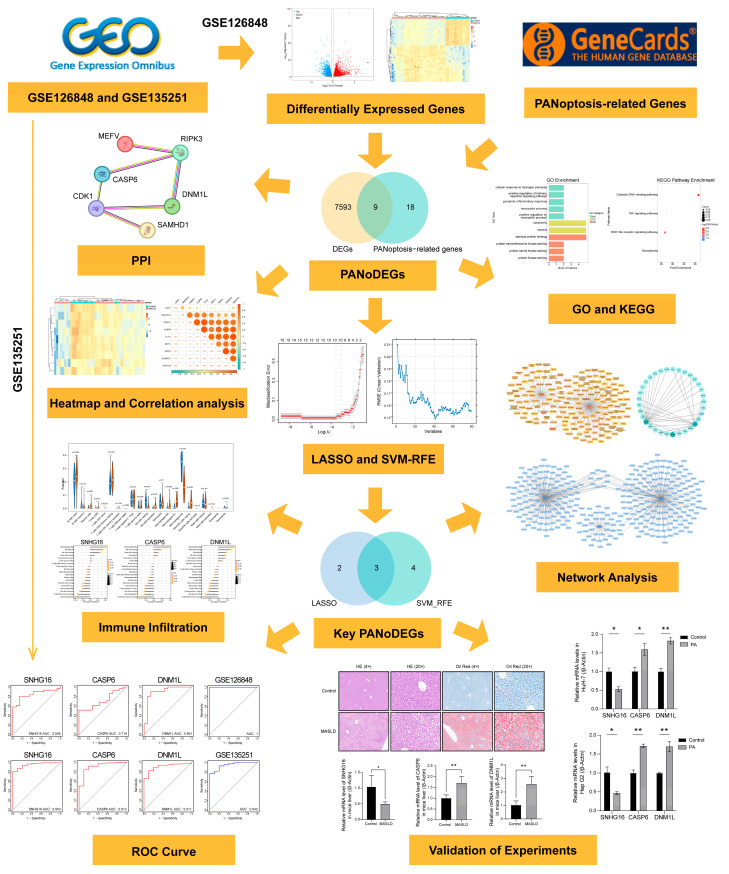
The key flowchart of this study. * *p* < 0.05, ** *p* < 0.01, *** *p* < 0.001.

In this study, we analyzed changes in gene expression profiles between 31 MASLD cases and 14 control cases sourced from the Gene Expression Omnibus (GEO) using bioinformatics techniques and identified differentially expressed genes associated with PANoptosis (PANoDEGs). Machine learning was employed to identify key PANoDEGs, and a receiver operating characteristic (ROC) curve analysis was performed to assess their diagnostic capability. Additionally, gene set enrichment analysis (GSEA) was employed to explore the single-gene enrichment of core PANoDEGs. In addition to examining immune infiltration in MASLD, we also investigated the immune characteristics of the key PANoDEGs. Furthermore, we explored the transcription factors, targeted microribonucleic acids (miRNAs), and targeted small-molecule drugs associated with key PANoDEGs. Additionally, we also established a regulatory network based on these findings. Finally, the expression of key PANoDEGs was validated using cellular, animal, and clinical sample experiments. The results of this study contribute to our understanding of the mechanistic role of PANoptosis in MASLD and identify promising therapeutic targets. Figure 1 summarizes the workflow of this study.

## 2. Materials and Methods

### 2.1. Data Acquisition

The GSE126848 dataset (platform: GPL18573) was downloaded from the GEO database (https://www.ncbi.nlm.nih.gov/geo/, accessed on 25 May 2024), including 31 MASLD liver samples (15 NAFL and 16 NASH) and 14 control samples. In addition, the GSE135251 dataset of human liver tissue containing 206 MASLD patients and 10 controls (platform: GPL18573) was downloaded from the GEO database and used as an external validation dataset for the critical PANoDEGs.

### 2.2. Identification of Differentially Expressed Genes (DEGs)

The GSE126848 dataset was normalized using the “DESeq2” package (version 1.38.3) to identify DEGs with statistically significant differences between the MASLD and normal samples (adjusted *p* < 0.05 and |fold change (FC)| > 1.2). Volcano plots and heatmaps were generated using the “ggplot2” (version 3.5.1) and “pheatmap” (version 1.0.12) packages, respectively.

### 2.3. PANoDEGs Identification

Genes of PANoptosis with a correlation score more than 1 were downloaded from the GeneCards database (Supplementary Table S2). These genes were then intersected with DEGs to obtain DEGs associated with PANoptosis (PANoDEGs), which was portrayed using a Venn diagram. Additionally, a correlation heatmap of PANoDEGs was generated using the “corrplot” package (version 1.0.12), while the “pheatmap” package was used to create a heatmap of PANoDEGs within the GSE126848 dataset.

### 2.4. Functional Enrichment Analysis

Gene Ontology (GO) and Kyoto Encyclopedia of Genes and Genomes (KEGG) pathway enrichment analyses of the PANoDEGs were performed in the DAVID database. The results of these enrichment analyses were illustrated using the “ggplot2” package in R.

### 2.5. Protein–Protein Interaction (PPI) Network Construction and Identification of Key PANoDEGs

The PPI network of the PANoDEGs was constructed and visualized in the STRING database using default parameters. In addition, the least absolute shrinkage and selection operator (LASSO) and support vector machine–recursive feature elimination (SVM-RFE) methods were applied to further filter the key PANoDEGs in the following analysis. Finally, the intersection of the results from the LASSO and SVM-RFE analyses was obtained to identify key PANoDEGs, which were represented visually through a Venn plot.

### 2.6. Diagnostic Values of the Key PANoDEGs in MASLD

The diagnostic performance of key PANoDEGs in distinguishing MASLD patients from controls was assessed using the area under the ROC curve (AUC). Additionally, they were further validated in the GSE135251 dataset. The “pROC” (version 1.18.5) package was used to depict the results.

### 2.7. Gene Set Enrichment Analysis (GSEA) for Key PANoDEGs

A total of 31 MASLD patients in the GSE126848 dataset were classified into low-expression and high-expression groups based on the median expression levels of key PANoDEGs. Then, the GSEA analysis was performed to analyze the functions enriched at different expression levels of each single gene.

### 2.8. Immune Infiltration Analysis

The CIBERSORT deconvolution algorithm was employed to analyze the immune cell subpopulation infiltration in the MASLD and control samples from the GSE126848 dataset. A Spearman rank correlation analysis was conducted to further explore the relationship between key PANoDEGs and immune cells. The results were visualized using the “ggplot2” package.

### 2.9. Network Analysis of the Key Genes

The Comparative Toxicological Genomics Database (CTD) is a public database that we used to explore interactions targeting critical PANoDEGs with counts of more than one potential therapeutic agent. The plug-in iRegulon (version 1.3) of Cytoscape (version 3.7.2) was applied to predict the transcription factors (TFs) of the key PANoDEGs. Finally, the combined TargetScan database and miRDB database were used to predict miRNA with potential interactions with key PANoDEGs (lncRNA predicts miRNA through the ENCORI database).

### 2.10. Cell Culture and Treatment

Hep G2 (CL-0103) and HuH-7 (CL-0120), provided by Wuhan Pricella Biotechnology Co., Ltd. (Wuhan, China), were cultured with a DMEM medium supplemented with 10% fetal bovine serum (Gibco, Grand Island, NY, USA). The cellular environment was strictly maintained at 37 °C, with 5% CO_2_. All cell lines were treated with palmitic acid (PA) (400 μM) (MedChemExpress, Monmouth Junction, NJ, USA) for 24 h.

### 2.11. Construction of an MASLD Animal Model

The animal procedures were approved by the Institutional Animal Care and Use Committee (Ethics approval number: HJSW-24051601) and strictly adhered to the “Guidelines for the Care and Use of Laboratory Animals”. Eight-week-old male C57BL/6J mice were divided into a control group and an MASLD group, with 4 mice in each group. Four mice were housed in each cage, and they were raised in the specific pathogen-free animal center of Jiangsu Hanjiang Biotechnology Co., Ltd. (Taizhou, China). The animal facility was maintained under a 12 h light/dark cycle, with the temperature controlled at 22 ± 2 °C and relative humidity at 50 ± 10%. The MASLD group was fed a high-fat diet (#D12492, Research Diets, New Brunswick, NJ, USA) with a caloric composition of 60% fat (lard and soybean oil), 20% protein (casein), and 20% carbohydrates (sucrose and maltodextrin), while the control group received a standard chow diet. After 12 weeks of feeding, the mice were euthanized, and their liver tissues were extracted. The relative values of ALT and AST in the mouse serum were determined by a biochemical analyzer.

### 2.12. Tissue Staining

The mouse liver tissues were fixed, dehydrated, and embedded in OCT compound, followed by cryosectioning at 5 μm thickness. These sections underwent hematoxylin–eosin (H&E) staining and Oil Red O staining, followed by careful photography for documentation. Five non-overlapping fields were selected from different parts of the hepatic lobule under low power, and the Oil Red O-positive area was measured by Image Pro Plus (Version 6.0, Media Cybernetics, Rockville, MD, USA) software for statistical analysis.

### 2.13. Clinical Samples Collected from MASLD Patients and Controls

The research protocol was authorized by the Medical Ethics Committee of the Affiliated Taizhou People’s Hospital of Nanjing Medical University (No: KY2022-192-01). The diagnosis of MASLD was based on chronic elevation of alanine aminotransferase (ALT) > 1.5 times the upper normal limit for more than 6 months, negative hepatitis B and hepatitis C markers, absence of autoimmune hepatitis, celiac disease, cholestatic liver diseases or cirrhosis, and no evidence of hereditary, drug-induced, or excessive alcohol consumption [20]. The patients with MASLD and the healthy controls were enrolled in this research. Written informed consent was obtained from all participants, permitting the use of their samples for analysis. This study complied with the ethical principles outlined in the Declaration of Helsinki. A total of 20 MASLD patients and 20 healthy individuals undergoing routine physical examinations were enrolled as the control group. Blood cells collected from all participants were obtained by isolating precipitates following centrifugation of whole blood at 3000 rpm for 15 min at 4 °C.

### 2.14. RNA Extraction and Quantitative Real-Time PCR (RT-qPCR)

Total RNA was extracted using an NcmSpin Cell/Tissue Total RNA Kit (#M5105, NCM, Suzhou, China) or RNAiso Blood (#9113, Takara, Shiga, Japan) and reverse transcribed into cDNA using a reverse transcription kit (#AG11728, AG, Changsha, Hunan, China). Gene expression profiles of SNHG16, CASP6, and DNM1L were assessed using a qPCR kit (#AG11733, AG, Changsha, Hunan, China) and LightCycler 480 II system (Basel, Switzerland). β-Actin was employed as an internal normalization control. All samples were processed in triplicate. Table 1 lists the primer sequences used in this study.

### 2.15. Statistical Analysis

Data analysis was performed using R software (version 4.2.3), and experimental data were validated with Prism 9.0. Data were presented as mean ± standard error of the mean (SEM). Differences were analyzed using an unpaired two-tailed Student’s *t*-test for two groups and analysis of variance (ANOVA) for more than two groups. *p* < 0.05 was considered statistically significant, unless otherwise specified.

## 3. Results

### 3.1. DEGs in MASLD

**Figure 2:**
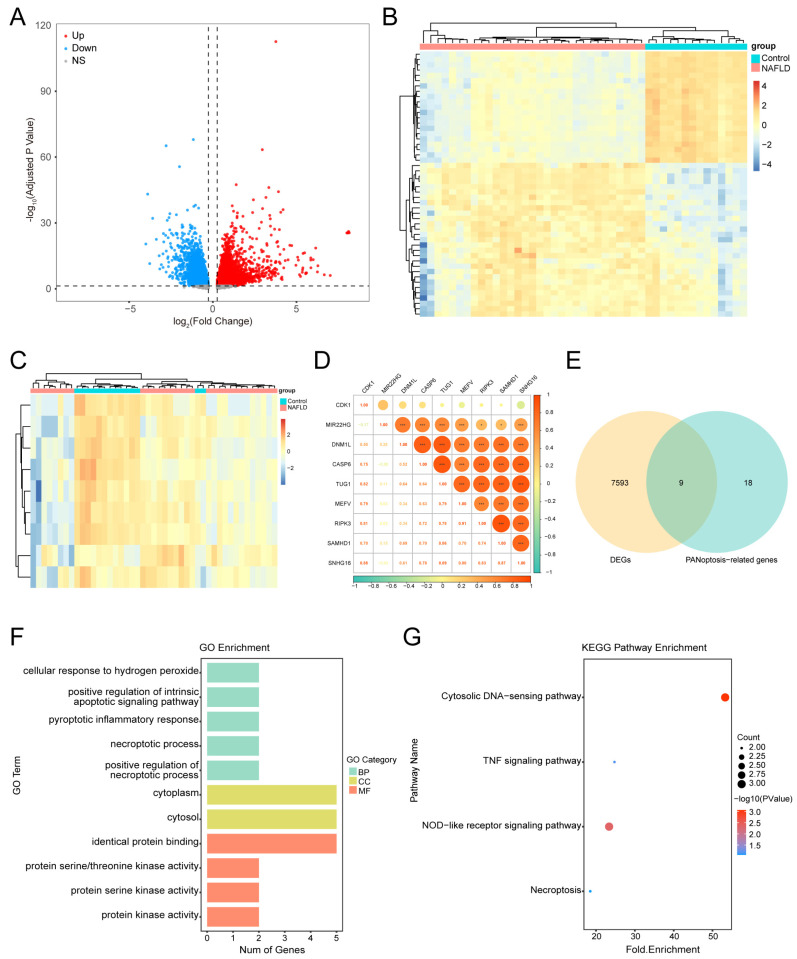
Identification of differentially expressed genes associated with PANoptosis (PANoDEGs) in nonalcoholic fatty liver disease and functional enrichment analysis. (**A**,**B**) Volcano plot and heatmap of DEGs in GSE126848. (**C**) Venn plots of the genes associated with DEGs and PANoptosis-related genes. (**D**) Heatmap of PANoDEGs in the GSE126848 dataset. (**E**) Heatmap of the correlation among the PANoDEGs genes. (**F**) Gene Ontology enrichment analysis of PANoDEGs, biological process, cellular component, and molecular function. (**G**) Kyoto Encyclopedia of Genes and Genomes pathway enrichment for PANoDEGs. * *p* < 0.05, *** *p* < 0.001.

A total of 7602 DEGs were identified in the GSE126848 dataset, with 4117 upregulated and 3485 downregulated genes (Supplementary Table S3). The volcano plot displays the DEGs between the MASLD and control samples (Figure 2A), and the heat map illustrates the top 50 DEGs ranked according to the adjusted *p*-value (Figure 2B).

### 3.2. Identifying PANoDEGs and Their Correlation

By considering the intersection of PANoptosis-related genes from the GeneCards database and DEGs, nine PANoDEGs were obtained, including four upregulated and five downregulated genes (Figure 2C) (Supplementary Table S4). The heatmap displays the expression of these PANoDEGs in the GSE126848 dataset (Figure 2D). The correlation heatmap suggested that these PANoDEGs were closely related to MASLD (Figure 2E).

### 3.3. Functional Enrichment Analysis of the PANoDEGs

The Gene Ontology (GO) functional enrichment analysis included 14 biological processes (BPs), two cellular components (CCs), and four molecular functions (MFs) (Supplementary Table S5). The GO enrichment analysis (Figure 2F) demonstrated that the PANoDEGs were significantly enhanced in intrinsic apoptosis, pyroptosis, necroptosis, identical protein binding, and protein kinase activity. According to the Kyoto Encyclopedia of Genes and Genomes pathway analysis, nine PANoDEGs were significantly enriched in four pathways, including the cytosolic DNA-sensing pathway, tumor necrosis factor (TNF) signaling pathway, NOD-like receptor signaling pathway, and necroptosis (Figure 2G, Supplementary Table S6).

### 3.4. PPI Network Analysis and Leveraging Machine Learning Identification of Key PANoDEGs

**Figure 3:**
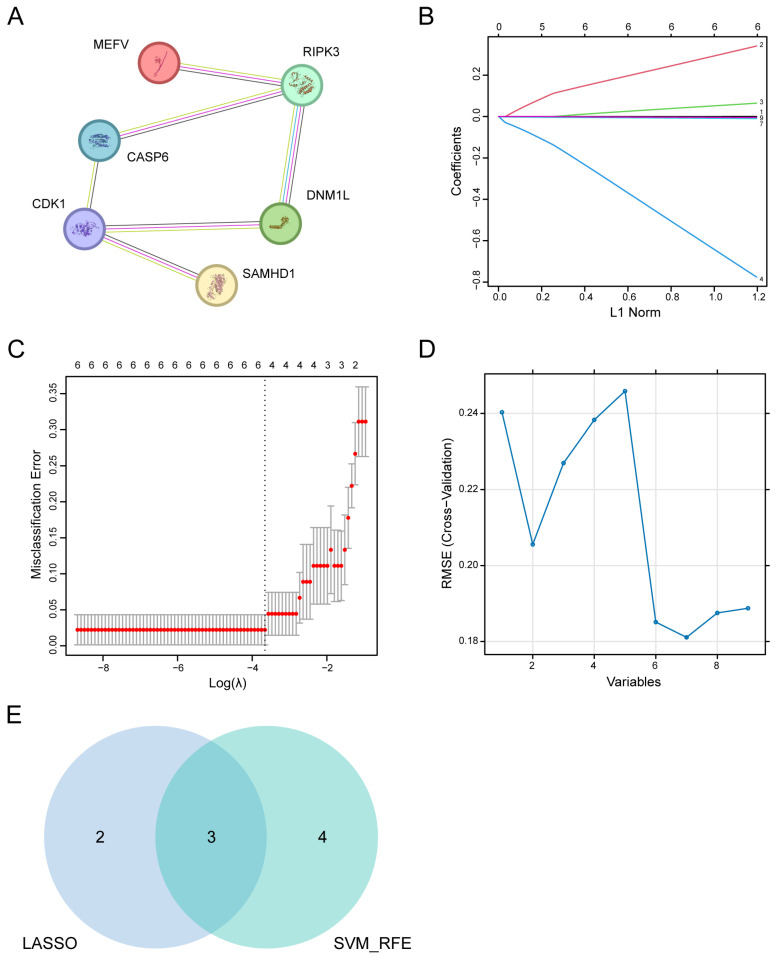
Identification of the key differentially expressed genes associated with PANoptosis (PANoDEGs) in nonalcoholic liver disease. (**A**) The protein–protein interaction network signature. (**B**,**C**) The Least Absolute Shrinkage and Selection Operator (LASSO) algorithm filtered the PANoDEGs. (**D**) PANoDEGs were prioritized through the SVM-RFE algorithm. (**E**) Venn diagram of critical PANoDEGs by LASSO and SVM-RFE. The shared selection of PANoDEGs by LASSO and SVM-RFE in the Venn diagram is designated as the key PANoDEGs.

We constructed a PPI network using the STRING database (confidence level > 0.4) to further investigate the interactions among the PANoDEGs (Figure 3A). In addition, in the machine learning algorithm, LASSO, identified five PANoDEGs by 10-fold cross-validation (Figure 3B,C), whereas SVM-RFE selected seven PANoDEGs (Figure 3D). Cross-referencing of these results yielded three key PANoDEGs (SNHG16, CASP6, and DNM1L) for further analysis (Figure 3E).

### 3.5. Clinical Diagnostic Significance of Key PANoDEGs for MASLD

**Figure 4:**
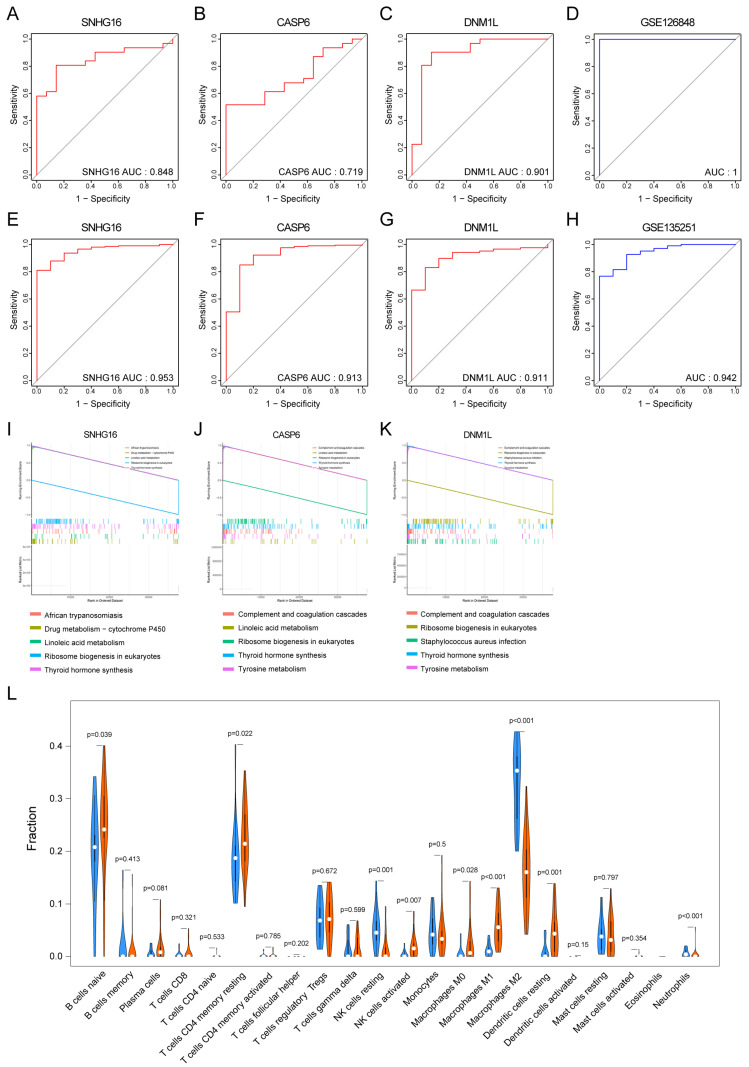
Diagnostic effect of key differentially expressed genes associated with PANoptosis (PANoDEGs), gene set enrichment analysis of key PANoDEGs, and immune infiltration. (**A**–**D**) Receiver operating characteristic (ROC) curves for assessing the diagnostic validity of critical PANoDEGs using the GSE126848 dataset. (**E**–**H**) ROC curves for the diagnostic validity of critical PANoDEGs evaluated using the GSE135251 dataset. (**I**–**K**) The five pathways with the highest enrichment scores including SNHG16, Caspase-6 (CASP6), and DNM1L. (**L**) Comparison of immune cell subtypes in nonalcoholic fatty liver disease and control liver, blue represents the control group, and red represents the disease group.

The ROC analysis suggested that key PANoDEGs exhibited significant diagnostic potential in distinguishing MASLD samples from control samples in the GSE126848 dataset. The area under the curve (AUC) for SNHG16, CASP6, and DNM1L was 0.848, 0.716, and 0.901, respectively (Figure 4A–C). The AUC of the combined predictive model for these three genes was 1 (Figure 4D). Further validation of the diagnostic abilities of the three genes in the GSE135251 dataset revealed that their AUCs, along with the AUC of the combined predictive model, were all above 0.9 (Figure 4E–H).

### 3.6. The GSEA of the Key PANoDEGs

Using the median expression of SNHG16, CASP6, and DNM1L as binding sites, 31 patients with MASLD were divided into low- and high-expression groups. GSEA was performed on SNHG16, CASP6, and DNM1L (Supplementary Table S7). The top five GSEA-related pathways of the key PANoDEGs were mainly concentrated in linoleic acid metabolism, thyroid hormone synthesis, tyrosine metabolism, and complement and coagulation cascades (Figure 4I–K).

### 3.7. Immune Infiltration Analysis for MASLD

Immune infiltration analysis indicated that the proportions of M1 macrophages, naïve B cells, and activated natural killer (NK) cells in MASLD tissues were significantly elevated compared with those in control tissues. However, the proportions of M2 macrophages, resting NK cells, and neutrophils were considerably reduced (Figure 4L).

**Figure 5:**
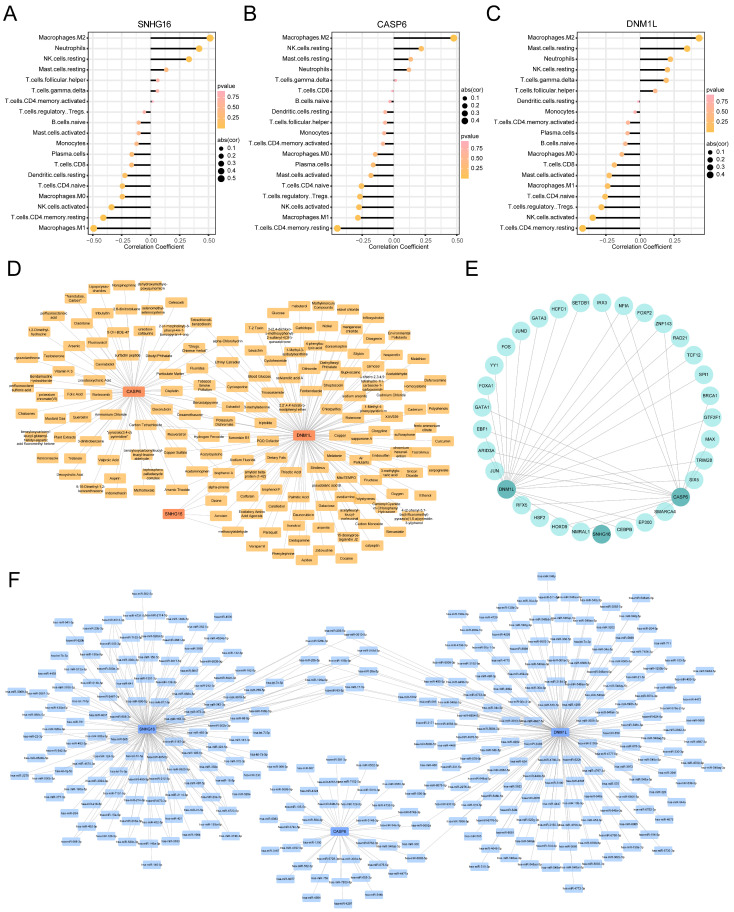
The immune infiltration analysis and network analysis of key differentially expressed genes associated with PANoptosis (PANoDEGs). (**A**) Correlation between SNHG16 and immune cells. (**B**) Correlation between CASP6 and immune cells. (**C**) Correlation between DNM1L and immune cells. (**D**) Key PANoDEGs–drug network analysis based on the CTD database. (**E**) Regulatory network of transcription factors and key PANoDEGs. (**F**) Microribonucleic acid and key PANoDEGs regulatory networks.

The correlation analysis between the key PANoDEGs in MASLD and infiltrating immune cells revealed that SNHG16 was positively correlated with M2 macrophages, neutrophils, and resting NK cells and negatively correlated with M1 macrophages, resting dendritic cells, and resting CD4+ memory T cells (Figure 5A, Supplementary Table S8). CASP6 positively correlated with CD8+ T cells, resting dendritic cells, naïve B cells, and M1 macrophages, but negatively correlated with neutrophils, resting NK cells, and follicular helper T cells (Figure 5B). Moreover, DNM1L was positively correlated with M1 macrophages, resting dendritic cells, and γδ T cells, while it was negatively correlated with M2 macrophages, naïve B cells, and resting NK cells (Figure 5C).

### 3.8. Targeted Drug Prediction for the Key PANoDEGs

Using the CTD database, we predicted a potential drug-target network for the key genes, revealing interactions between SNHG16, CASP6, DNM1L, and 167 different small molecules as well as drugs (Figure 5D, Supplementary Table S9).

### 3.9. TFs and miRNA Network Analysis of the Key PANoDEGs

We expanded our analysis to explore the inter-regulatory relationships between key genes and upstream regulatory factors. Specifically, we focused on the interactions of these genes with a network of 39 TFs (Figure 5E) (Supplementary Table S10) and their connections with 317 miRNAs (Figure 5F) (Supplementary Table S11). However, it is important to note that these potential regulatory networks require further verification.

### 3.10. Experimental Validation of the Critical PANoDEGs

**Figure 6:**
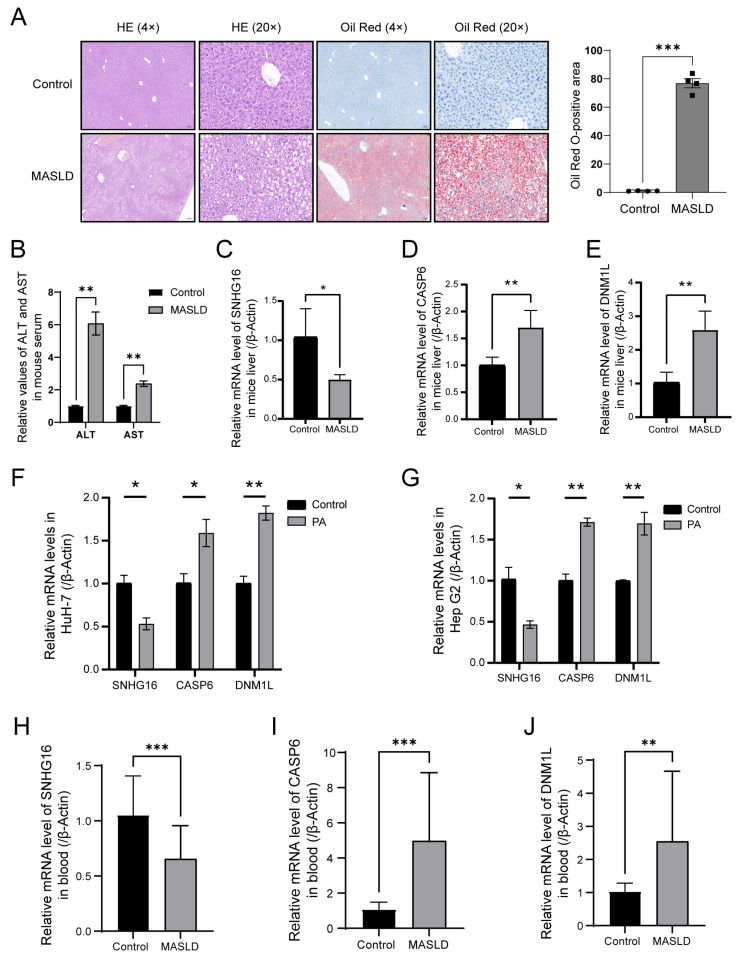
Validation of the expression of key differentially expressed genes associated with PANoptosis (PANoDEGs) in MASLD mouse models and cells. (**A**) Hematoxylin–eosin and Oil Red O staining of the control and MASLD livers of mice; bar (4×) = 200 μm and bar (20×) = 50 μm. (**B**) The relative values of ALT and AST in mouse serum. (**C**–**E**) Reverse transcription quantitative polymerase chain reaction (RT-qPCR) analysis of key PANoDEGs messenger ribonucleic acid (mRNA) levels in the control and MASLD livers of mice. (**F**,**G**) RT-qPCR analysis of key PANoDEGs mRNA levels of the control and PA-treated cells. (**H**–**J**) Results of the RT-qPCR analysis performed to measure the mRNA levels of key PANoDEGs in the blood samples obtained from the control group and MASLD group. * *p* < 0.05, ** *p* < 0.01, *** *p* < 0.001.

In the animal experiments, hematoxylin–eosin (HE) and Oil Red O staining demonstrated significant hepatic steatosis and sporadic inflammation in the MASLD group (Figure 6A). The results of the biochemical analyzer also showed that ALT and AST in the serum of the MASLD group were significantly higher than those of the control group (Figure 6B). All these results suggested that there was liver injury in the MASLD group. Consistent with human transcriptome data, the messenger RNA (mRNA) expression of the three key PANoDEGs revealed that the expression of SNHG16 in the MASLD group was significantly lower than that in the control group (*p* < 0.05) (Figure 6C), whereas the expression of CASP6 and DNM1L in the MASLD group was higher than that in the control group (*p* < 0.05) (Figure 6D,E). We further investigated the mRNA expression of the three key PANoDEGs in an MASLD cell model. The expression of CASP6 and DNM1L was significantly increased, whereas that of SNHG16 was significantly reduced in the PA-treated HuH-7 and Hep G2 cells (*p* < 0.05) (Figure 6F,G). Furthermore, 20 patients were enrolled in the MASLD group, and 20 healthy participants were enrolled in the control group. The mRNA expression of CASP6 and DNM1L increased significantly, whereas that of SNHG16 significantly decreased in the MASLD group (*p* < 0.05) (Figure 6H–J).

## 4. Discussion

MASLD is recognized as the most common form of chronic liver disease, often progressing to severe stages, leading to cirrhosis, end-stage liver disease, and hepatocellular carcinoma. Moreover, individuals with MASLD are at elevated risk of cardiovascular events, diabetes, and renal impairment, contributing to substantial public health challenges [21,22,23]. However, the intricate mechanisms underlying MASLD are poorly understood. Additionally, effective clinical treatment strategies are currently lacking. Thus, further investigation of the potential mechanisms and new molecular targets for effective therapy is essential.

Increasing evidence suggests that PCD is crucial in the development and progression of MASLD [5,6,7,8]. Recent studies indicated that Si-Wu-Tang alleviates PANoptosis in hepatocytes by blocking mitochondrial DNA release, which depends on the interaction between NOXA/Pmaip1 and VDAC2 [24]. However, the role of PANoptosis in MASLD remains poorly understood. To date, no bioinformatics studies have highlighted MASLD-related PANoptosis genes. In this study, we employed bioinformatic techniques to identify potential PANoptosis in MASLD. By intersecting the DEGs with PANoptosis-related genes, we obtained a set of nine PANoDEGs associated with MASLD. Enrichment analyses revealed that these genes were involved in intrinsic apoptosis, pyroptosis, necroptosis, cytoplasmic DNA sensing, TNF signaling pathways, and NOD-like receptor signaling. Using machine learning, we identified three key PANoDEGs and validated their diagnostic relevance in MASLD.

SNHG16 is a long-chain non-coding RNA closely associated with apoptosis. Studies have indicated that SNHG16 knockdown can inhibit the proliferation of various malignant tumors, including renal clear-cell carcinoma, colorectal cancer, neuroblastoma, and osteosarcoma, while promoting apoptosis [25,26,27,28]. Notably, the expression of SNHG16 is significantly elevated in liver cancer tissues and cell lines, where it exerts oncogenic effects through pathways such as miR-17-5p/p62, miR-4500/STAT3, and miR-605-3p/TRAF6/NF-κB [29,30,31]. Additionally, SNHG16 may contribute to sorafenib resistance in hepatocellular carcinoma via the EGFR1/miR-23b-3p and miR-140-5p pathways [32,33]. However, the mechanisms of action of SNHG16 in MASLD are yet to be reported. We observed significant downregulation of SNHG16 expression in both the livers of MASLD animals and clinical blood samples. Whether inhibition of the expression of SNHG16 mediates PANoptosis in MASLD through the aforementioned pathways requires further investigation.

CASP6 was first identified as an executor of apoptosis. Scientists have unveiled the ability of CASP6 as a regulator of cell apoptosis, playing a significant role in the host’s immune response to influenza A virus infection. Furthermore, CASP6, through its interaction with RIPK3, promotes the binding of the RIPK3-dependent RIP homotypic interaction motif to ZBP1, thereby facilitating ZBP1-mediated inflammasome activation, cell death, and host defense [34,35]. Consistent with this, our key PANoDEGs, including CASP6, are essential features of PANoptosis in MASLD. Interestingly, some studies suggest that Fibroblast Growth Factor 4 may have a protective effect against nonalcoholic steatohepatitis (NAS) by suppressing the cleavage and activation of CASP6 via the AMP-activated protein kinase (AMPK) signaling pathway [36]. Additionally, the literature indicates that bisalicylate was demonstrated to reverse metabolic disturbances in an MASLD mouse model by activating AMPK and inhibiting CASP6 activity [37]. Our animal and cell models indicated that CASP6 levels were elevated in the MASLD group, and the same trend was observed in the blood samples from the patients with MASLD. These findings suggest that CASP6 plays a role in MASLD apoptosis. However, whether CASP6 mediates hepatocyte apoptosis in MASLD via inflammatory complexes, including RIPK3, requires further research.

DNM1L, also referred to as Dynamin-related protein 1, is a critical protein involved in regulating mitochondrial dynamics. Research has indicated that, under conditions of pathologically high intraocular pressure, which is a hallmark of glaucoma, DNM1L induces mitochondrial dysfunction, leading to PANoptosis in retinal ganglion cells [38]. Additionally, DNM1L is also potentially involved in PANoptosis in atherosclerosis [19]. Although the regulatory mechanism of DNM1L in MASLD has not been reported, studies have suggested that DNM1L can alleviate NAS by reducing endoplasmic reticulum stress, preventing Oma1 activation, and mitigating integrated stress response deterioration [39]. Conversely, other studies have proposed that DNM1L plays a negative regulatory role in MASLD. These findings suggest that silencing DNM1L and/or chemically inhibiting DNM1L using mitochondrial division inhibitor 1 significantly impedes mitochondrial fission and improves lipid droplet accumulation induced by bisphenol F (BPF) in mouse and human liver cells induced by BPF [40]. Zhong et al. discovered that diosgenin mitigated mitochondrial fission–fusion barriers by inhibiting DNM1L and increasing MFN 1/MFN 2 expression, thereby enhancing fatty acid oxidation and mitochondrial function in type 2 diabetes-related MASLD [41]. Furthermore, Hu et al. noted that aerobic exercise regulated the acetylation of DNM1L and inhibited its activity by activating Sirtuins1, thereby alleviating MASLD and mitochondrial dysfunction [42]. These results suggest that DNM1L plays a crucial role in the development of MASLD, with its mediated mitochondrial dysfunction potentially serving as a key component in triggering PANoptosis in MASLD. However, these findings warrant further investigation.

MASLD pathophysiology is associated with immune inflammation [43]. Therefore, we thoroughly investigated the immune infiltration present in MASLD, along with the correlation between the expression of the three essential PANoDEGs and immune infiltrating cells. Importantly, macrophages are the dominant immune/inflammatory cells in MASLD tissues, with resident liver macrophages (Kupffer cells) and circulating recruited macrophages central to disease progression [44,45,46]. Different T-cell subtypes also significantly contribute to either supportive or antagonistic effects in the advancement of MASLD [47,48,49]. Furthermore, key PANoDEGs had connections with B cells, the most prevalent lymphocytes in the liver, where the antibodies and cytokines produced by B cells are vital for the pathogenesis of MASLD [50,51]. Moreover, these key PANoDEGs were linked to NK cells, consistent with the outcomes of previous reports of NK cell involvement in liver injury, regeneration, and fibrotic responses. Therefore, this suggests that NK cell damage may underpin the progression of fibrosis [52]. Overall, these results suggest that the mechanisms of key PANoDEGs in specific immune cell subtypes are complex and require further investigation to confirm their roles in MASLD.

Notably, we conducted a detailed examination of the expression profiles of key PANoDEGs in animal and cell models of MASLD. HE and Oil Red O staining demonstrated prominent hepatic steatosis in the MASLD mouse model. Moreover, reverse transcription quantitative polymerase chain reaction experiments revealed that the mRNA levels of CASP6 and DNM1L were significantly increased in the liver tissues of the MASLD mice compared to the levels in the controls, whereas SNHG16 exhibited the opposite trend. Additionally, these mRNA expression patterns were further corroborated in the PA-treated HuH-7 and Hep G2 cell lines. The expression patterns of the three key PANoDEGs were verified in the blood samples of patients with MASLD.

However, our study has certain limitations. Firstly, the causal relationship between the PANoDEGs identified in this study and MASLD progression remains unclear and requires further investigation to be elucidated. Especially in different disease progression stages, we lack in-depth exploration of the role of PANoDEGs in this process. In the follow-up, we need to focus on analyzing the specific mechanisms of PANoDEGs in different stages of MASLD, especially in the NASH stage. Secondly, validation of the three key genes selected is currently limited to their expression in cells, animal models, and clinical blood samples, which may potentially overlook other important molecular pathways related to PANoptosis in the progression of MASLD. These findings need to be confirmed in clinical pathological tissues, and further exploration of the underlying molecular mechanisms is required. Thirdly, this study relied heavily on bioinformatic tools and a limited number of datasets, which may have introduced potential biases and limitations. For instance, demographic differences or disease severity may have influenced participant variability. These potential biases may restrict the generalizability of our findings to a broad population. However, our findings offer a potential starting point for future research on PANoptosis in MASLD, offering insights into novel diagnostic strategies and therapeutic targets for this condition. These findings warrant further exploration and validation in large, well-characterized cohorts.

## 5. Conclusions

Overall, this study investigated the gene expression profiles and immune cell infiltration landscapes in both MASLD and control groups, highlighting the potential role of SNHG16, CASP6, and DNM1L in PANoptosis during the immune/inflammatory response in MASLD. Additionally, we created the first comprehensive regulatory network of key genes linked to PANoptosis in MASLD, revealing the underlying mechanisms involved in MASLD and offering clues for novel diagnostic and therapeutic strategies.

## Figures and Tables

**Table 1:** Primer sequences used in RT-qPCR.

Gene	Species	Forward (5′–3′)	Reverse (5′–3′)	Gene Function (Brief Description)
SNHG16	Human	AGAGACCAAGGAGGGACTGT	TACTGGCACGAGGACAAAGC	SNHG16 (Small Nucleolar RNA Host Gene 16) is a long non-coding RNA. It is involved in regulating gene expression and is associated with processes such as cell proliferation and apoptosis. It may play an important role in diseases like MASLD.
	Mouse	ATCATGGAAAGGCGTGGTGG	ATCTGCCACTTAGCACACCC	
CASP6	Human	GCAGATGCCGATTGCTTTGT	GTCTCCTTTGAACAAGCCAGTTA	CASP6 (Caspase-6) is a cysteine protease. It plays a crucial role in apoptosis and the inflammatory response by cleaving specific substrate proteins, thereby promoting the process of programmed cell death.
	Mouse	CATGACGTACCCGTGGTTCC	AGCCATTCACAGTTTCTCGGT	
DNM1L	Human	AGTGGTGACTTGTCTTCTTCGTAA	TAGCCTGTTTCTCCTTTGTTCCT	DNM1L (Dynamin-1-like protein) participates in intracellular organelle dynamic changes such as mitochondrial fission. It is of great significance for maintaining the normal morphology and function of mitochondria within cells, and its abnormal function may be related to various diseases.
	Mouse	CCATTATCCTCGCCGTCACT	GCATCAGTACCCGCATCCAT	
β-Actin	Human	CATGTACGTTGCTATCCAGGC	CTCCTTAATGTCACGCACGAT	β-Actin is a constitutively expressed cytoskeletal protein. It is widely present in eukaryotic cells and is often used as an internal reference gene for gene expression analysis. It is involved in various physiological processes of cells, such as cell movement and shape maintenance.
	Mouse	GGCTGTATTCCCCTCCATCG	CCAGTTGGTAACAATGCCATGT	

## Data Availability

The data in this study are available to the public in the Gene Expression Omnibus (GEO) database (GSE126848 and GSE135251). In addition, GeneCards (https://www.genecards.org, accessed on 25 May 2024) collects PANoptosis genes.

## Supplementary Materials

The following supporting information can be downloaded at: https://www.mdpi.com/article/10.3390/biology14050518/s1, Supplementary Table S1: The characteristics of programmed cell death. Supplementary Table S2: PANoptosis related genes obtained from the GeneCards database; Supplementary Table S3: Differentially expressed genes and their calculated values in the GSE126848 dataset; Supplementary Table S4: 9 PANoptosis-related differentially expressed genes; Supplementary Table S5: The GO functional enrichment analysis results of 9 PANoDEGs; Supplementary Table S6: The KEGG pathway analysis results of 9 PANoDEGs; Supplementary Table S7: Gene set enrichment analysis of key PANoDEGs; Supplementary Table S8: The correlation between key PANoDEGs and infiltrated immune cells; Supplementary Table S9: Drug network analysis of key PANoDEGs; Supplementary Table S10: Prediction of TFs networks for key PANoDEGs; Supplementary Table S11: Prediction of miRNAs networks for key PANoDEGs.

## Abbreviations

The following abbreviations are used in this manuscript:

MASLD	metabolic dysfunction-associated steatotic liver disease
NAFLD	non-alcoholic fatty liver disease
MASH	metabolic dysfunction-associated steatohepatitis
NASH	previously known as nonalcoholic steatohepatitis
PCD	programmed cell death
AIM2	absent in melanoma 2
ZBP1	Z-nucleic-acid binding domain protein 1
ASC	apoptosis-associated speck-like protein containing a C-terminal CARD
RIPK3	receptor-interacting serine/threonine-protein kinase 3
mtDNA	mitochondrial DNA
DEGs	differentially expressed genes
PANoDEGs	differentially expressed genes associated with PANoptosis
ROC	receiver operating characteristic
GEO	gene expression omnibus
LASSO	least absolute shrinkage and selector operation
SVM-RFE	support vector machine–recursive feature elimination
Limma	Linear Model of Microarray Data
PPI	protein–protein interaction
GO	Gene Ontology
KEGG	Kyoto Encyclopedia of Genes and Genomes
BP	biological process
CC	cellular component
MF	molecular function
STRING	Search Tool for the Retrieval of Interacting Genes
AUC	area under curve
GSEA	gene set enrichment analysis
CTD	comparative toxicogenomics database
TFs	transcription factors
RT-qPCR	real-time quantitative polymerase chain reaction
SNHG16	small nucleolar RNA host gene 16
CASP6	Caspase-6
DNM1L	Dynamin-1-like protein
DRP1	Dynamin-related protein 1
PA	palmitic acid
