# Supplementary material for: Comprehensive Analysis Reveals the Molecular Features and Immune Infiltration of PANoptosis-Related Genes in Metabolic Dysfunction-Associated Steatotic Liver Disease
Source: Biology (Basel). 2025 May 8;14(5):518. doi: 10.3390/biology14050518 (PMC12108815; doi:10.3390/biology14050518)
Supplement: Supplementary file 1 [file biology-14-00518-s001.zip › Supplementary Table S1. The characteristics of programmed cell death.pdf]

Supplementary Table S1: The characteristics of programmed cell death.

Subtype	Definition and Characteristics	Key Molecules	Morphology
Apoptosis	Occurs via two main pathways. The extrinsic one starts when extracellular ligands bind death receptors, activating caspase - 8. The intrinsic pathway is triggered by intracellular stress, causing mitochondrial outer membrane permeabilization and cytochrome c release. Both activate downstream caspases for apoptosis execution.	Death receptors, caspase - 8, Bcl - 2 family, cytochrome c, Apaf - 1, caspase - 9, 3, 7	Cell shrinkage, chromatin condensation, nuclear fragmentation, apoptotic body formation
Pyroptosis	A pro - inflammatory form of programmed cell death mediated by the activation of inflammasomes and the cleavage of gasdermin D by caspase - 1 or caspase - 11 (in mice)	Caspase - 1, caspase - 11 (in mice), gasdermin D, NLRP3 inflammasome, AIM2 inflammasome	Cell swelling, rapid membrane rupture, release of pro - inflammatory cytokines (IL - 1β, IL - 18)
Necroptosis	A regulated form of necrosis that occurs when apoptosis is inhibited, mainly mediated by the RIPK1 - RIPK3 - MLKL pathway	RIPK1, RIPK3, MLKL	Cell swelling, membrane rupture, causing inflammation
PANoptosis	A regulated inflammatory cell death pathway that combines features of apoptosis, necroptosis, and pyroptosis, mediated by the formation of a PANoptosome complex	Sensors (ZBP1, NLRP3, AIM2), adapters (ASC, FADD), catalytic effectors (RIPK1, RIPK3, CASP1, CASP8, MLKL, Gasdermins)	Cell swelling, membrane rupture, and in some cases, chromatin condensation
